# A T-DNA mutant screen that combines high-throughput phenotyping with the efficient identification of mutated genes by targeted genome sequencing

**DOI:** 10.1186/s12870-019-2162-7

**Published:** 2019-12-04

**Authors:** Ulrike Frank, Susanne Kublik, Dörte Mayer, Marion Engel, Michael Schloter, Jörg Durner, Frank Gaupels

**Affiliations:** 1 Institute of Biochemical Plant Pathology, Helmholtz Zentrum München, Neuherberg, Germany; 2 Research Unit for Comparative Microbiome Analysis, Helmholtz Zentrum München, Neuherberg, Germany; 3 Scientific Computing Research Unit, Helmholtz Zentrum München, Neuherberg, Germany; 4 Chair of Biochemical Plant Pathology, Technische Universität München, Freising, Germany

**Keywords:** *Arabidopsis thaliana*, T-DNA insertion, Mutant screen, High-throughput phenotyping, Target enrichment, Next generation sequencing, Adapter ligation-mediated PCR

## Abstract

**Background:**

Nitrogen dioxide (NO_2_) triggers hypersensitive response (HR)-like cell death in *Arabidopsis thaliana*. A high-throughput mutant screen was established to identify genes involved in this type of programmed cell death.

**Results:**

Altogether 14,282 lines of SALK T-DNA insertion mutants were screened. Growing 1000 pooled mutant lines per tray and simultaneous NO_2_ fumigation of 4 trays in parallel facilitated high-throughput screening. Candidate mutants were selected based on visible symptoms. Sensitive mutants showed lesions already after fumigation for 1 h with 10 ppm (ppm) NO_2_ whereas tolerant mutants were hardly damaged even after treatment with 30 ppm NO_2_. Identification of T-DNA insertion sites by adapter ligation-mediated PCR turned out to be successful but rather time consuming. Therefore, next generation sequencing after T-DNA-specific target enrichment was tested as an alternative screening method. The targeted genome sequencing was highly efficient due to (1.) combination of the pooled DNA from 124 candidate mutants in only two libraries, (2.) successful target enrichment using T-DNA border-specific 70mer probes, and (3.) stringent filtering of the sequencing reads. Seventy mutated genes were identified by at least 3 sequencing reads. Ten corresponding mutants were re-screened of which 8 mutants exhibited NO_2_-sensitivity or -tolerance confirming that the screen yielded reliable results. Identified candidate genes had published functions in HR, pathogen resistance, and stomata regulation.

**Conclusions:**

The presented NO_2_ dead-or-alive screen combined with next-generation sequencing after T-DNA-specific target enrichment was highly efficient. Two researchers finished the screen within 3 months. Moreover, the target enrichment approach was cost-saving because of the limited number of DNA libraries and sequencing runs required. The experimental design can be easily adapted to other screening approaches e.g. involving high-throughput treatments with abiotic stressors or phytohormones.

## Background

Forward genetics by mutant screens is a widely used approach to experimentally assign biological functions to genes or characterize physiological processes through the identification of involved genes [1]. Thousands of mutant lines were generated in *Arabidopsis thaliana*, *Oryza sativa*, *Solanum lycopersicum*, *Medicago truncatula*, and other plant species by insertional mutagenesis of genes using retrotransposons or *Agrobacterium tumefaciens*-derived T-DNA [2–5]. For Arabidopsis, the SALK collection represents the largest source of T-DNA insertion mutants covering almost the complete genome [1]. The fact that the T-DNA sequence is known facilitates convenient mapping of the insertion site. For instance, several PCR-based methods such as the adapter ligation-mediated PCR rely on insert-specific primers to selectively amplify the T-DNA/genomic DNA junction before identification of the mutated gene by sequencing [6]. Alternatively, insert specific biotinylated 70mer probes can be employed for the isolation of T-DNA-containing DNA fragments by streptavidin beads [7]. Combining this target enrichment with next generation sequencing (NGS) allowed the simultaneous identification of multiple insertion sites in a complex pool of DNA from as much as 64 different mutants [7]. Both, adapter ligation-mediated PCR and target enrichment followed by NGS (i.e. targeted genome sequencing) are suitable for high-throughput mappings of mutation sites [6, 7].

Nitrogen dioxide (NO_2_) is a notorious toxic air pollutant but also an upcoming endogenous signal in plant cells where it arises from the oxidation of NO and nitrite or decomposition of peroxynitrite [8, 9]. Fumigation experiments revealed that NO_2_ has beneficial as well as detrimental effects on plant performance dependent on the applied gas concentration. Short-term treatments of Arabidopsis for 1 h with 10 ppm NO_2_ did not cause visible leaf damage but triggered transient salicylic acid (SA) accumulation and basal pathogen resistance against *Botrytis cinerea* and *Pseudomonas syringae* [10]. Treatments of Arabidopsis seedlings for 8 h with 10 ppm or 1 h with 30 ppm NO_2_ caused apparent lesion formation [11, 12]. NO_2_-induced cell death was reminiscent of the hypersensitive response (HR) of resistant plants towards avirulent pathogens, which ultimately cumulates in programmed cell death (PCD) [13–15]. NO_2_-induced cell death – like HR-PCD – was found to be dependent on simultaneous signaling by H_2_O_2_ and NO, and was enhanced in SA deficient mutants [11, 12, 16, 17]. Moreover, both cell death events were accompanied by the accumulation of fluorescent phenolic compounds (Additional file 1: Figure S1) and oxylipins including jasmonates [11]. Collectively, these findings suggest that NO_2_ exposure leads to the dose-dependent induction of basal pathogen resistance and HR-like cell death. How NO_2_ exerts these different effects in plants remains ambiguous.

Therefore, a high-throughput genetic screen was developed to identify new genes involved in NO_2_ sensitivity and -tolerance. Altogether 14,282 individual lines of SALK T-DNA insertion mutants were screened for their NO_2_-induced leaf symptoms. Sensitive mutants displayed lesions already after fumigation with 10 ppm NO_2_ whereas the other tested plants were unaffected. Tolerant mutants survived even exposure to 30 ppm NO_2_ without severe damage. Subsequently, T-DNA-mutated genes were identified by adapter ligation-mediated PCR or genome sequencing after T-DNA-specific target enrichment. The latter technique proved to be particularly efficient facilitating the identification of 162 genes with putative functions in NO_2_ sensitivity or tolerance. An initial re-screen of corresponding mutants confirmed that 8 of 10 tested candidate mutants were altered in NO_2_-induced leaf damage as compared to wild-type (WT) plants.

Overall, the current paper describes an experimental set-up that facilitates the efficient genome-wide screening, identification and investigation of T-DNA insertion mutants. The experimental design can be adapted to other high-throughput treatments e.g. with abiotic stressors or phytohormones. Although the screen was optimized for Arabidopsis, it would also be possible to screen insertion mutants in other plant species.

### Challenges/tasks

Whole genome screen ➔ many mutant lines must be handled

NGS ➔ large datasets must be handled ➔ advanced strategy for data handling/filtering required

Screen should be cost-, time, and labor-saving

Advantages and disadvantages of published screens and mutant collections

## Results

### High-throughput screening for NO_2_-sensitive and -tolerant T-DNA insertion mutants

**Fig. 1:**
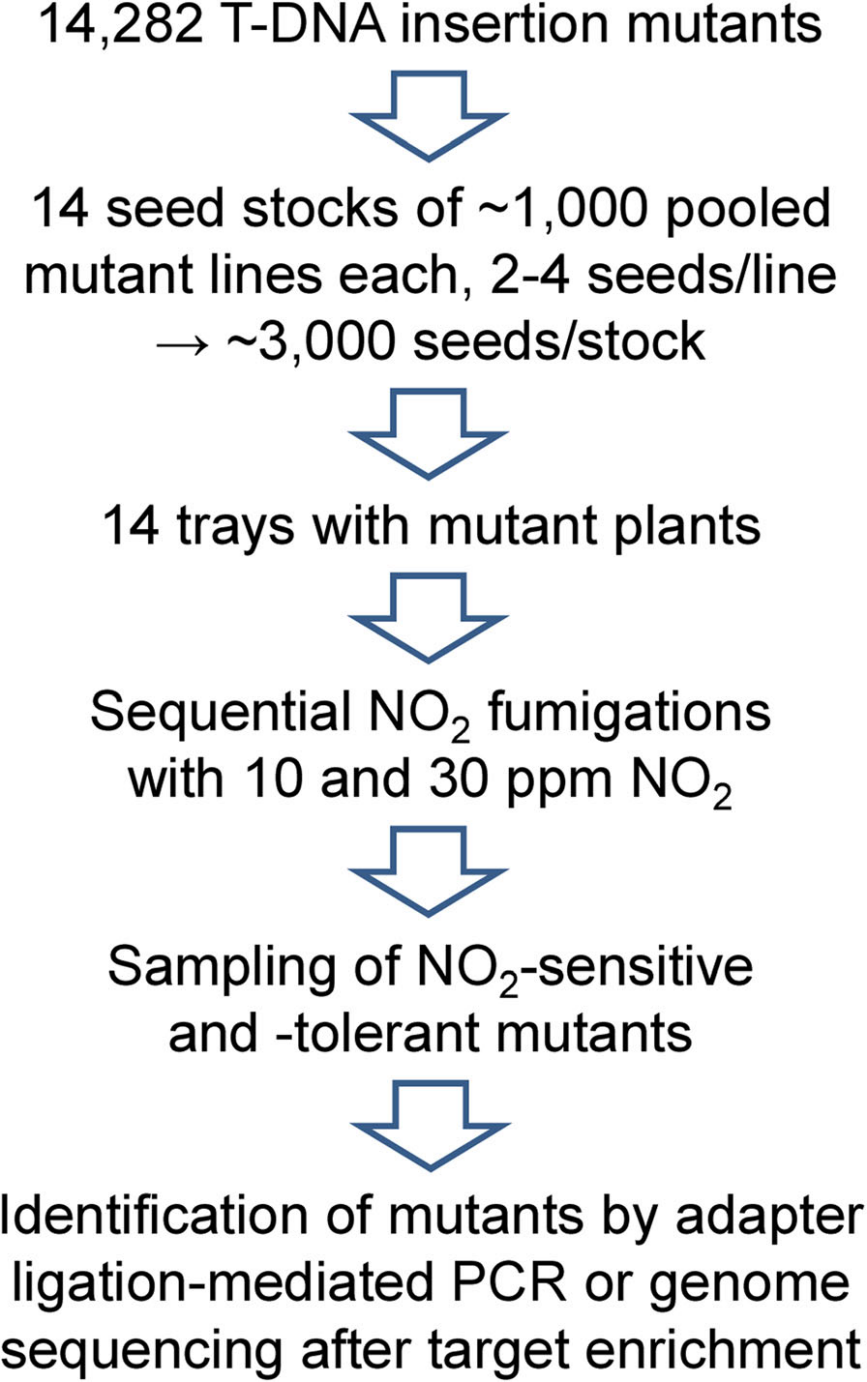
Strategy of the NO_2_ screen of Arabidopsis T-DNA insertion mutants

**Fig. 2:**
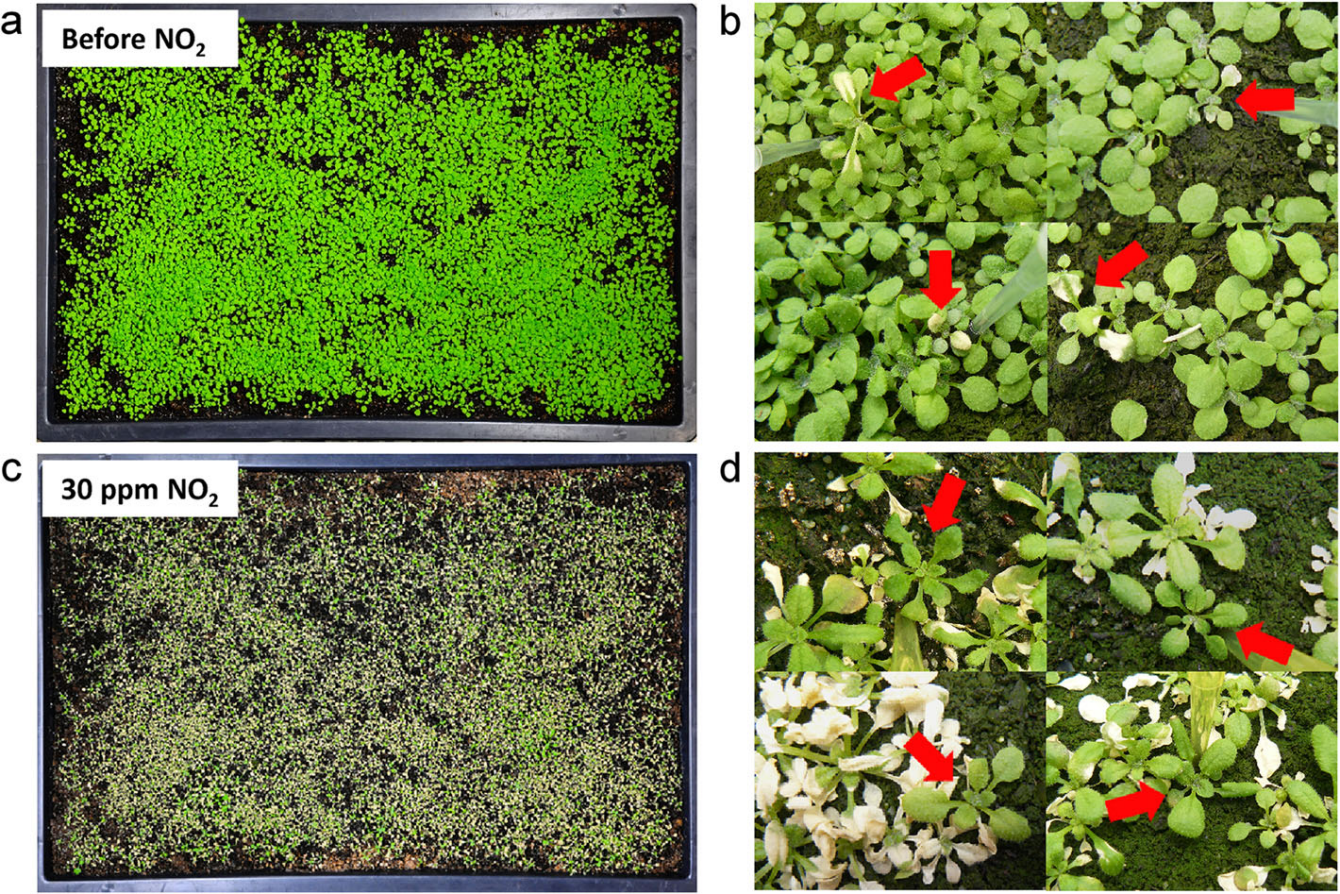
Detection of NO_2_-sensitive and -tolerant mutants. **a.** Approximately 3000 mutant plants per tray were grown for 2 weeks. Plants were fumigated for 1 h with 10 ppm NO_2_ and 2 days later with 30 ppm NO_2_. **b** Example of a sensitive mutant showing lesions after fumigation with 10 ppm NO_2_ while neighbouring plants were unaffected. Pictures were taken at 48 h after fumigation. **c** Most plants were severely damaged after exposure to 30 ppm NO_2_. **d** However, tolerant mutants exhibited only weak symptoms. Pictures were taken at 48 h – 96 h after fumigation. Note that the highlighted (red arrows) mutants exhibited altered NO_2_-induced symptoms compared to all neighboring plants

Altogether 14,282 SALK T-DNA insertion mutants (“confirmed homozygous” mutant sets) were screened for their sensitivity or tolerance towards NO_2_. In order to limit the investment of hands-on time and climate chamber space the different mutant lines were not grown separately but were pooled (Fig. 1). Each ~ 1000 mutant lines (2–4 seeds per line) were combined resulting in only 14 trays of plants (Fig. 1, Fig. 2a). Fourteen-day-old mutant seedlings were short-term fumigated for 1 h with 10 ppm NO_2_ and (2 days later) 30 ppm NO_2_. The exposure chamber allowed NO_2_ treatment of 4 trays in parallel, i.e. fumigation of all 14 trays with one concentration of NO_2_ took only 4 h. Sensitive plants developed lesions already after exposure to 10 ppm NO_2_ whereas the other plants were not affected (Fig. 2b). Subsequent treatment with 30 ppm NO_2_ caused severe leaf damage in almost all plants (Fig. 2c) whereas tolerant mutants displayed only weak symptoms (Fig. 2d). Altogether, 124 SALK T-DNA insertion lines showing distinct NO_2_ phenotypes were sampled and DNA was extracted.

### Identification of mutated genes by adapter ligation-mediated PCR

Adapter ligation-mediated PCR is the standard method employed by the SALK institute to define T-DNA insertion sites [6]. It is based on digestion of the DNA by the restriction enzyme *Ase*1, Ase adapter ligation, and selective amplification of T-DNA-containing fragments using the T-DNA left border-specific primer LBb1 and the adapter-specific primer AP2 before sequencing. Hence, adapter ligation-mediated PCR promotes the targeted amplification and sequencing of T-DNA/genomic DNA junctions. Here, this approach was tested with three NO_2_-tolerant candidate mutants detected in the screen.

**Fig. 3:**
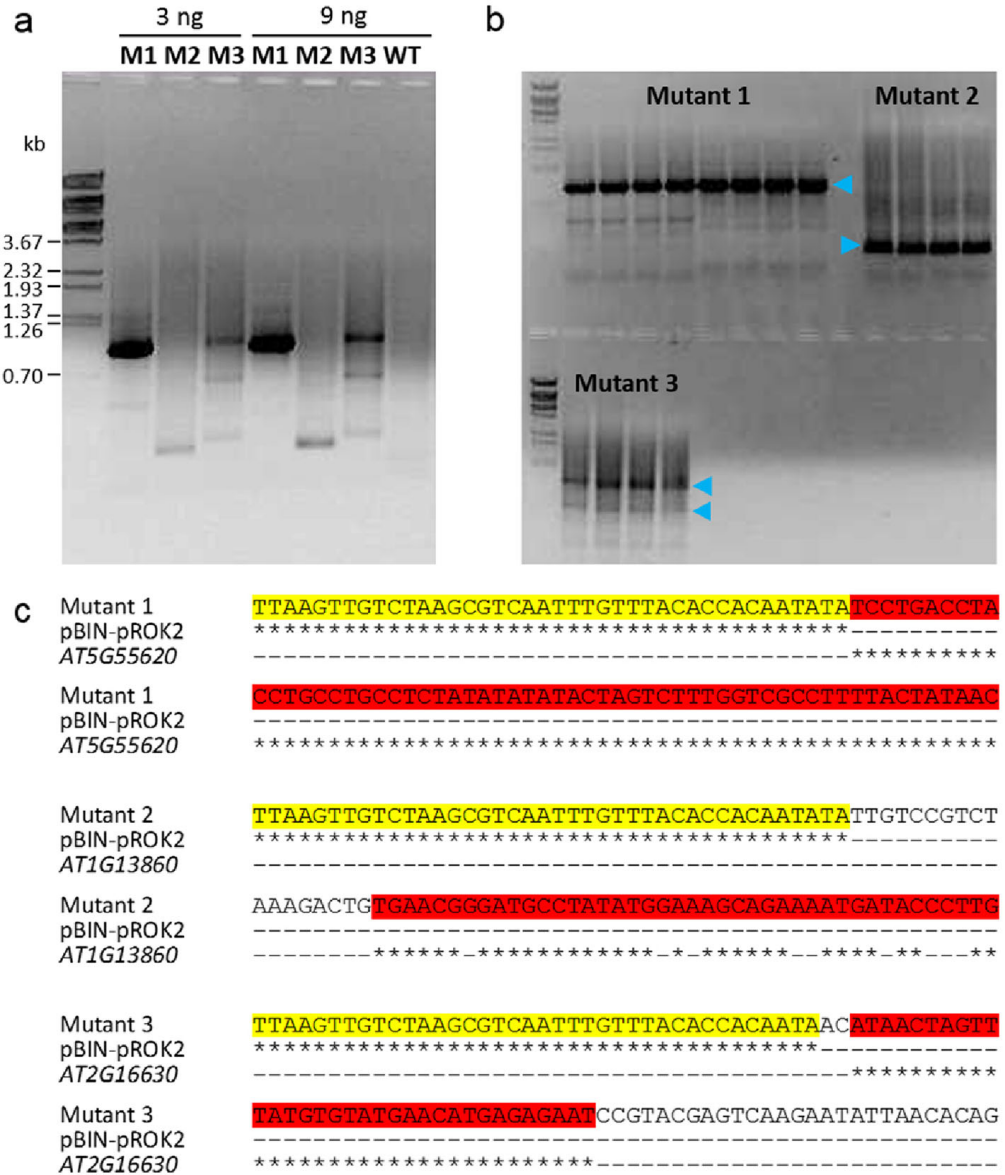
Identification of candidate genes by adapter ligation-mediated PCR. **a** DNA of the mutants 1, 2, and 3 (M1, M2, M3) was extracted and T-DNA containing fragments were selectively amplified by nested PCR. DNA of a wild-type plant (WT, Col-0) served as T-DNA negative control. For each mutant line 3 and 9 ng DNA was loaded on an agarose gel. DNA standards are indicated. **b** Multiple PCR reactions (9 ng DNA per lane) were loaded on an agarose gel to obtain sufficient amounts of DNA for sequencing. Major bands (blue arrow heads) were cut, and the eluted DNA was sequenced. **c** Results of sequencing, homology search and candidate gene alignment are shown. For mutant 3 both cut bands contained DNA with the displayed consensus sequence. Homology of mutant DNA to the pBIN-pROK2 vector (T-DNA) or an Arabidopsis gene is highlighted in yellow and red, respectively. Asterisks indicate matching bases. Only 100 bases around the T-DNA-gene junctions are shown. BLAST hit for Mutant 1: NM_124944.4, *AT5G55620*, E value 0.0, 100% identities (726/726). BLAST hit for Mutant 2: CP002684.1, *AT1G13860*, *QUL1*, E value 0.0, 89% identities (527/592). BLAST hit for Mutant 3: NM_127215.5, *AT2G16630*, *FOCL1*, E value 2e-06, 100% identities (33 of 33). The T-DNA-flanking genomic sequences used for the BLAST searches are given in Additional file 2: Table S1

Selective amplification of the T-DNA-containing fragments and separation in an agarose gel resulted in a clear band for mutant 1 but weaker bands for mutants 2 and 3 (Fig. 3a). The low DNA concentrations in the excised bands prevented successful sequencing. Therefore, multiple PCR reactions were separated in an agarose gel, and the major band for mutant 1 and mutant 2 was cut (Fig. 3b). Furthermore, the two strongest bands for mutant 3 were analyzed to check whether they represent two independent T-DNA insertion sites (Fig. 3b). Sequencing of the eluted DNA revealed that in all cases the investigated DNA fragments contained the T-DNA left border (Fig. 3c). The DNA from mutant 1 additionally included a 726 bases-long sequence with 100% homology to the Arabidopsis gene *AT5G55620*. For mutant 2 the 592 bases adjacent to the T-DNA border shared significant homology with the gene *AT1G13860*/*QUASIMODO-LIKE1*/*QUL1*. Both analyzed mutant 3 bands contained a short DNA stretch of only 33 bases next to the T-DNA border that was homologous to the gene *AT2G16630*/*FUSED OUTER CUTICULAR LEDGE1*/*FOCL1*. The bases adjacent to this sequence did not result in a BLAST (Basic Local Alignment Search Tool) hit. Hence, both analyzed bands for mutant 3 represented the same T-DNA insertion. In sum, adapter ligation-mediated PCR successfully identified T-DNA insertion sites in all three investigated mutants.

### Identification of mutated genes by genome sequencing after T-DNA target enrichment

**Fig. 4:**
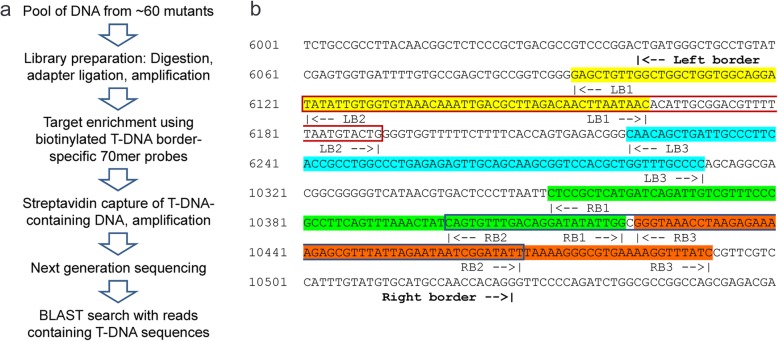
The T-DNA target enrichment approach. **a** Overview of the experimental procedure. **b** Sequences of the T-DNA border-specific 70mer probes used for target enrichment. Partial sequences of the pBIN-pROC2 vector containing the T-DNA left and right borders are shown. Binding sites of the 70mer probes specific for the T-DNA left border (LB1 (yellow background color), LB2 (red frame), LB3 (blue background)) and right border (RB1 (green background), RB2 (blue frame), RB3 (orange background)) are indicated

In a second approach (Fig. 4a) DNA aliquots of the 124 sampled mutant seedlings were combined in two pools each consisting of 1 μg DNA from 59 or 65 mutant seedlings. The preparation of the two libraries involved digestion of the DNA, adapter ligation, and amplification. At this stage T-DNA-containing DNA fragments were hidden within the complex total DNA pools. Therefore, 6 T-DNA border-specific biotinylated 70mer probes served to semi-purify the T-DNA-containing DNA fragments (Fig. 4a and b) [7]. Successful purification was confirmed by quantitative polymerase chain reaction (qPCR) with T-DNA-specific primers before identification of the T-DNA insertion sites by next generation sequencing. It is important to note that the target enrichment approach required processing of only two DNA pools and only two runs of next generation sequencing.

High proportions of long reads (400–700 bases) and Phred scores around 30 (i.e. 99.9% probability of a correct base call) indicated a good sequencing quality for both libraries. Further analysis of the sequencing results revealed that in both libraries approx. 75% of the reads contained T-DNA proving again the successful T-DNA enrichment (Table 1). By comparison, in another study based on target enrichment 40% of the reads mapped to the T-DNA [7]. More stringent filtering criteria were applied to extract high-quality reads as a pre-requisite for the correct determination of T-DNA insertion sites. The raw datasets were filtered by BLASTN searches against the T-DNA containing vector pBIN-pROK2 with high-scoring segment pair (HSP) length set to be between 30 and 343 bp and an Expect (E) value threshold of below 5.72*E^− 11^. This procedure removed > 97% of the total sequencing reads (Table 1). Additional trimming of the data for read length (> 40 base pairs (bp)) and quality (low quality limit 0.05, ambiguous bases ≤2) as well as disposal of adapter-only- and vector-only sequences reduced the number of remaining reads to 264 for library I and 374 for library II corresponding to 0.1 and 0.2% of the original sequencing reads, respectively. 255 (97%) and 367 (98%) of these reads mapped to the Arabidopsis genome suggesting that the bioinformatic processing of the datasets yielded meaningful results (Table 1).

**Table 1:** Filtering of sequencing reads improves mapping to the Arabidopsis genome

	Library I		Library II	
	No. of reads	%	No. of reads	%
Total sequencing reads	207,785	100.0	154,847	100.0
1. Filter: T-DNA containing reads	163,166	78.5	116,160	75.0
2. Filter^a^: HSP length, e-value	1536	0.7	3932	2.5
3. Filter^b^: Read length and quality, removal of adapter-only and vector-only reads	264	0.1	374	0.2
Mapped to the Arabidopsis genome	255	0.1	367	0.2

^a^ BLASTN search against the pBIN-PROK2 sequence with the settings: High-scoring segment pair (HSP) length ≥ 30 bp to ≤343 bp, E value ≤5.72*E^−11^; ^b^ read length > 40 bp, low quality limit 0.05, ambiguous bases ≤2, removal of the 5′ terminal nucleotide, removal of adapter-only and vector-only sequences

The altogether 622 reads mapped to 162 Arabidopsis genes. All 3 T-DNA insertion sites defined by adapter ligation-mediated PCR were also detected by the target enrichment approach which can be interpreted as internal cross-validation of both methods. Although *AT2G16630*/*FOCL1* had a T-DNA insertion as shown by both approaches, the corresponding mutants were not present in the SALK mutant collection used for the screen. A second site mutation of *AT2G16630* seems unlikely because adapter ligation-mediated PCR detected only a single T-DNA insertion. More likely, the used seed stocks included an *AT2G16630* mutant that was incorrectly annotated or labelled. For instance, the SALK_042357 line has a T-DNA mutation in the position determined by the targeted genome sequencing (Additional file 3: Figure S2) but was not included in the screened mutant sets. Another remarkable finding of the target enrichment approach was that the characterization of 124 NO_2_-sensitive and -tolerant mutant seedlings led to the identification of 162 T-DNA-containing genes. This could be related to unnoticed secondary T-DNA insertions in the tested mutants or experimental artefacts such as sequencing errors. Seventy mutated genes were identified by ≥3 reads, 23 genes by 2 reads, and 69 genes by 1 read. The most solid candidate genes were selected for further characterization based on a read number ≥ 3 and presence of the corresponding mutant line in the screened mutant collection.

### Confirmation of the NO_2_-induced mutant phenotypes by a re-screen

A re-screen was initiated to confirm the altered NO_2_-induced cell death phenotypes of the identified candidate mutants. Visible symptoms are not a suitable estimate of cell death because they can be influenced e.g. by altered leaf toughness or enhanced chlorosis in certain mutants. Therefore, cell death was quantified by ion leakage measurements after fumigation for 40 min with 30 ppm NO_2_. In addition, the basal stomatal conductance, which is a determining factor of NO_2_ uptake via the stomatal pores, was assessed in untreated leaves using a porometer [11]. The 10 tested mutants were identified by ≥3 reads in the target enrichment approach (Table 2). Additionally, the mutants *qul1* and *at5g55620* were also identified by adapter ligation-mediated PCR.

**Table 2:** Description of re-screened candidate mutants

Mutated gene	Gene name	Reads	SALK line
*AT4G15090*	*FAR1*	4	SALK_031652C
*AT3G62600*	*ERDJ3B*	10	SALK_055599C
*AT1G13860*	*QUL1*	15	SALK_048823C
*AT2G45560*	*CYP76C1*	10	SALK_010566C
*AT1G49910*	*BUB3.2*	4	SALK_151687C
*AT1G04930*	*AT1G04930*	14	SALK_015636C
*AT5G55620*	*AT5G55620*	5	SALK_018370C
*AT5G17420*	*CESA7*	3	SALK_029940C
*AT1G52810*	*AT1G52810*	5	SALK_050959C
*AT3G13235*	*DDI1*	6	SALK_066713C

**Fig. 5:**
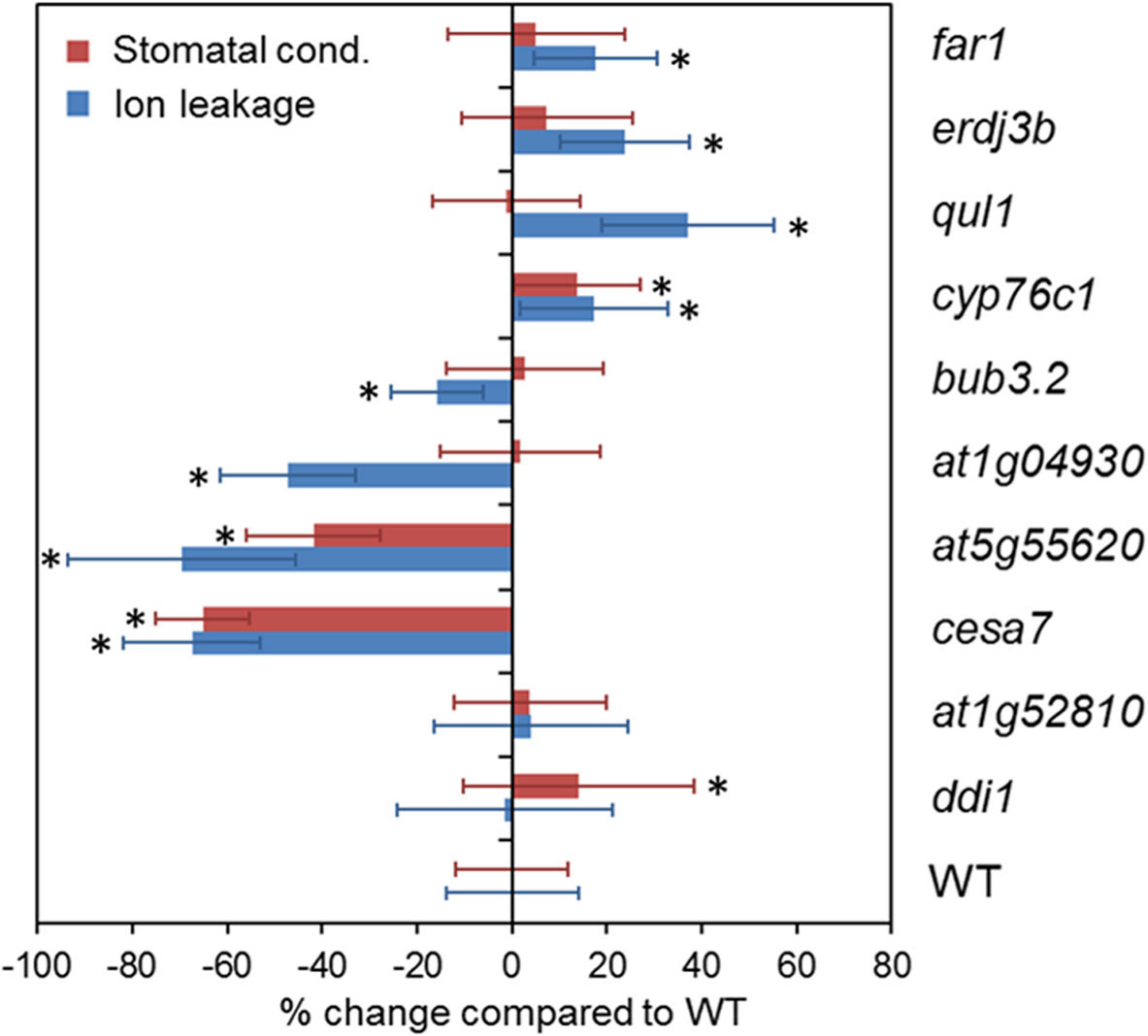
Re-screen of candidate mutants. Ten mutants defective in genes identified by the target enrichment approach were re-screened. The genes *AT1G13860* (*QUL1*) and *AT5G55620* were also identified by adapter ligation-mediated PCR. The tested mutants were not genotyped. Ion leakage was determined upon fumigation with 30 ppm NO_2_ whereas stomatal conductance was measured in untreated plants. Asterisks indicate statistically significant differences (*p* ≤ 0.05) between WT and tested mutants according to one-way ANOVA and Holm-Sidak posthoc test (ion leakage: *n* = 15–28; stomatal conductance: *n* = 12–23)

All tested mutants had a basal ion leakage similar to WT confirming that they did not develop spontaneous lesions (Additional file 4: Figure S3a). Eight of 10 investigated mutants showed different extents of HR-like cell death after NO_2_ fumigation compared to WT plants (Fig. 5; Additional file 4: Figure S3b and c). The mutants *far1*, *erdj3b*, *qul1*, and *cyp76c1* were sensitive but *bub3.2*, *at1g04930*, *at5g55620*, and *cesa7* rather tolerant to NO_2_. *qul1* was found to be tolerant in the original screen but sensitive in the re-screen. The reason for this inconsistency is unknown. Two mutants had an enhanced stomatal conductance phenotype (Fig. 5). For *cyp76c1* the increased stomatal uptake of NO_2_ correlated with stronger symptom development whereas *ddi1* showed similar symptoms like WT. By contrast, the strong NO_2_ tolerance of *at5g55620* and *cesa7* was linked to reductions of the stomatal conductance by 42 and 65%, respectively. Three mutants exhibited elevated and 2 mutants decreased NO_2_-induced ion leakage but no stomata phenotype. Ion leakage was 18–37% higher in *far1*, *erdj3b*, and *qul1* but 16 and 47% lower in *bub3.2* and *at1g04930* compared to WT (Fig. 5).

**Fig. 6:**
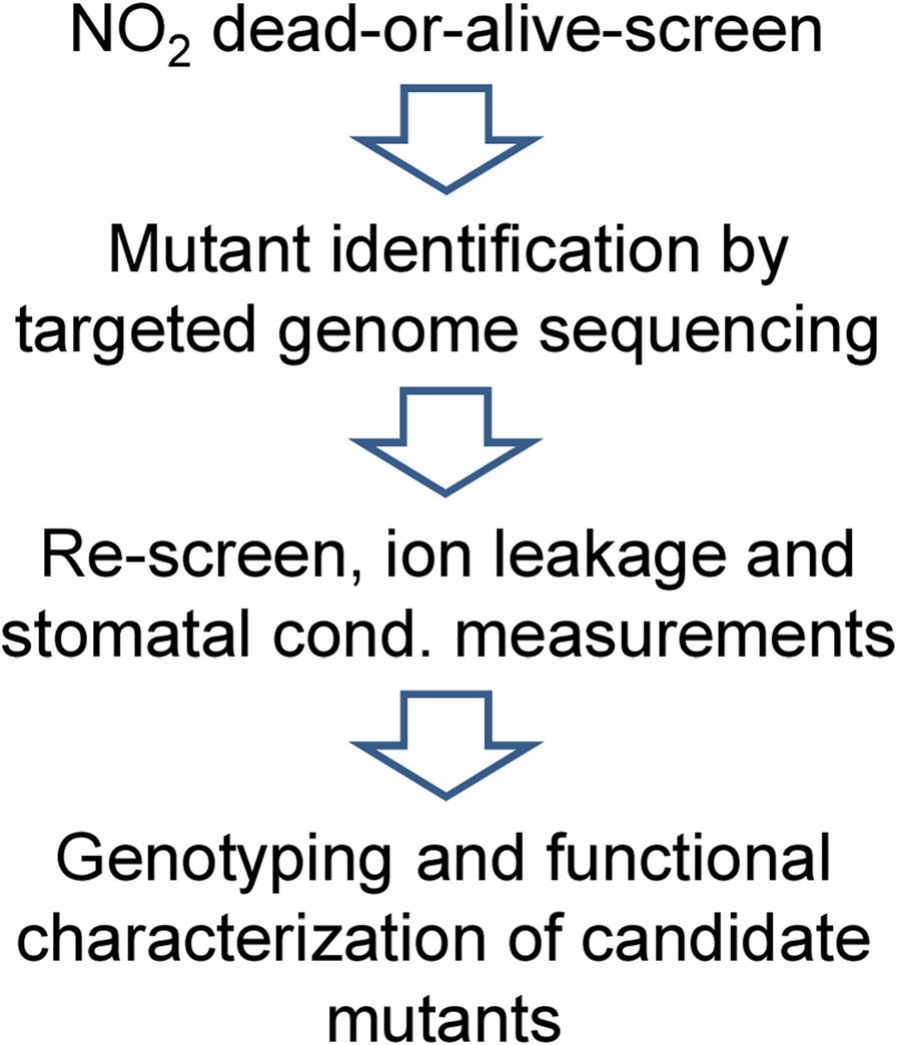
Summary of the T-DNA mutant screen. Genotyping and functional characterization of candidate mutants represents future work not described in the current report

The NO_2_-sensitive or -tolerant mutants described above are candidates for further characterization. Although SALK mutants with confirmed T-DNA insertion sites were purchased for the study, homozygosity of the mutants must be confirmed by genotyping (Fig. 6). After the re-screen, genotyping will be necessary only for the most promising mutants. Mutant backcrossing or the investigation of two independent mutant lines per candidate gene prevents artefacts e.g. due to unnoticed secondary T-DNA insertions. Ultimately, it will be investigated whether knockout- and over-expressor lines of selected genes show specific phenotypes related to NO_2_-induced cell death, HR-PCD and pathogen resistance.

## Discussion

The project aimed at establishing an efficient genome-wide screen of Arabidopsis mutants to identify genes involved in NO_2_ sensitivity and tolerance. The mutant collection investigated so far comprised 14,282 SALK T-DNA insertion lines, where altogether 10,849 individual genes were mutated. Hence, the collection covered more than one third of the complete Arabidopsis genome. High-throughput screening was facilitated by pooling of each ~ 1000 mutant lines (~ 3000 seeds) resulting in only 14 trays of mutant seedlings to be handled (Fig. 1). Growing the plants on soil within a climate chamber prevented exposure of the plants to artificial conditions prevailing e.g. in petri dishes or 96-well plates. Additionally, this set-up allowed the efficient treatment of many mutant lines in parallel. Other main features of the screen included short-term NO_2_ fumigations for only 1 h, NO_2_ treatments of 4 trays in parallel, and simple selection of candidate mutants for their visible NO_2_ phenotypes (Fig. 2). This way, 124 candidate mutants displaying distinct NO_2_ phenotypes were collected. A single person could carry out the NO_2_ screen within one-month period.

Adapter ligation-mediated PCR identified T-DNA insertion sites in all three investigated mutants (Fig. 3). However, at least in our hands and without robotics the method was suitable only for low numbers of mutants because it was rather time consuming. Moreover, costly enzymes, chemicals and sequencing were required for every single mutant analysis. By contrast, targeted genome sequencing required the processing of just 2 libraries containing the pooled DNA of all 124 candidate mutants (Fig. 4). The identification of mutated genes further involved target enrichment using T-DNA border-specific 70mer probes (Fig. 4), next generation sequencing, and stringent filtering of the sequencing reads by bioinformatic tools (Table 1). This strategy led to the identification of 70 T-DNA insertion sites by at least 3 sequencing reads. It is important to note that all 3 gene mutations detected by adapter ligation-mediated PCR were also found by targeted genome sequencing indicating that both approaches yielded similar results. Overall, the one-by-one analysis of mutants by adapter ligation-mediated PCR reduced the risk that a mutant remained unidentified without notice whereas NGS after target enrichment had the advantage of being time and cost saving.

Ten mutants defective in identified candidate genes were re-screened. Eight of these mutants showed altered NO_2_-induced cell death compared to WT plants confirming that results of the screen were reproducible. The NO_2_ sensitivity of *cyp76c1* mutant correlated with an enhanced leaf uptake of NO_2_ due to an increased stomatal conductance whereas the reduced stomatal conductance of *cesa7* and *at5g55620* probably accounted for the strong NO_2_ tolerance of these mutants (Fig. 5). The CELLULOSE SYNTHASE 7-deficient mutant *cesa7* is disturbed in xylem-mediated water transport, which triggers a constitutive down-regulation of the stomatal aperture to reduce water loss due to transpiration [18]. Moreover, inhibition of the cell wall biosynthesis in *cesa7* leads to enhanced abscisic acid-dependent resistance against *Ralstonia solanacearum* [19]. *AT5G55620* is an ethylene-responsive gene that codes for an uncharacterized protein [20]. Functions of CYTOCHROME P450 76C1 (CYP76C1) are also unknown. However, the data presented here argue for a role of AT5G55620 and CYP76C1 in stomatal regulation. It will be interesting to learn whether pathogen-induced HR-PCD is altered in *cyp76c1*, *at5g55620*, and *cesa7*.

Other mutants showed a distinct NO_2_- but no stomata phenotype (Fig. 5). Hence, the respective genes code for proteins that probably function in cell death induction or protection rather than stomata regulation. *far1*, *erdj3b*, and *qul1* were sensitive but *bub3.2* and *at1g04930* tolerant towards NO_2_. The transcription factor FAR-RED IMPAIRED RESPONSE 1 (FAR1) prevents light-induced oxidative stress and SA-dependent cell death [21], and thus might also protect cells from NO_2_-induced cell death and HR-PCD. ERDJ3B is a chaperone involved in the assembly of the immune receptor kinase EFR [22]. Its role in basal pathogen resistance could be connected to cell death protection by unknown mechanisms. The ubiquitin ligase BUB3.2 acts in cell division. *BUB3.2* gene expression was down-regulated in Arabidopsis pistils after infection with the fungal pathogen *Fusarium graminearum* [23]. The fact that ion leakage is decreased in the knockout mutant argues for BUB3.2 being a positive regulator of NO_2_-induced HR-like cell death. *qul1* showed 37% increased but *at1g04930* 47% decreased ion leakage after NO_2_ fumigation compared to the WT. Functions of the mutated genes were not yet investigated. Therefore, the current results represent initial evidence that the genes could be involved in cell death regulation.

NO_2_ triggers basal pathogen resistance or HR-like cell death in a dose-dependent manner [10, 11]. Accordingly, the results described here suggest that the NO_2_ dead-or-alive screen is a useful tool to identify genes related to HR (−like)-PCD, pathogen resistance, and stomata regulation.

## Conclusions

The described experimental system allows for the efficient screening and identification of Arabidopsis T-DNA insertion mutants. Main features of the NO_2_ dead-or-alive screen included (1.) the growth and treatment of 1000 pooled mutant lines per tray and (2.) candidate mutant selection based on obvious damage symptoms. Main features of the mutant identification by the target enrichment approach included (1.) pooling of DNA from ~ 60 candidate mutants per library, (2.) T-DNA-specific target enrichment before next-generation sequencing, and (3.) stringent filtering of the sequencing reads. Mutant identification by adapter ligation-mediated PCR might be advisable only if robotics is available. A re-screen revealed that 8 of ten tested mutants showed NO_2_ phenotypes thereby confirming that the results of the screen were reliable. The described experimental system can be applied for any screening of Arabidopsis T-DNA insertion mutants based on high-throughput treatments inducing obvious phenotypes. Moreover, the screening procedure could even be adapted to insertion mutants in other plant species such as rice and tomato.

## Methods

### Plants and growth conditions

SALK T-DNA insertion mutants were used in this study. Generation, formal identification, and submission of the mutant lines to public seed stocks was done by the SALK Institute Genomic Analysis Laboratory (SIGnAL) [24]. The four sets of confirmed homozygous lines with the NASC (Nottingham Arabidopsis Stock Centre) order numbers N27941, N27951, N27942, and N27952 comprised altogether 14,282 T-DNA mutants. All lines are available from NASC (http://arabidopsis.info). Fourteen seed pools of approx. 1000 mixed mutant lines each were prepared. To this end, 2–4 seeds per line were flipped out of every mutant stock tube by a bended needle forming a little hook. Thus, one seed pool contained approx. 3000 seeds, and in sum > 42,000 seeds were used in the screen. Fourteen plant trays (size: 0.6 m × 0.4 m × 0.06 m) with inlets (tray-sized, holes in the bottom to prevent water logging) were filled with soil (4 parts Floradur propagation substrate (Floragard) mixed with 1 part quartz sand). Seeds of every pool were evenly spread in each tray and vernalized for 2 days at 4 °C in the dark.

### NO_2_ fumigations

The mutant screen was done in walk-in size chambers housing 4 air tight fumigation chambers (www.helmholtz-muenchen.de/eus/facilities/phytotron), which allowed simultaneous fumigation of 4 plant trays in parallel. The growth conditions were set to 250 μmol m^− 2^ s^− 1^ light intensity, 14 h light-10 h dark cycle, 23 °C/18 °C (day/night), and 70%/90% (day/night) relative humidity. The plants were grown for 2 weeks in the chambers, and mutants exhibiting chlorosis or lesions were removed before the NO_2_ treatments. NO_2_ was generated by the reaction of 15% NO with 100% O_2_ in mixing vessels containing Raschig glass rings. The NO_2_ concentrations were adjusted by regulating the NO flux rate. The concentrations of NO_2_ were monitored with an AC3 2 M chemiluminescent oxides of nitrogen analyzer (Environnement S.A.). Generally, the fumigations started in the morning at approx. 2 h after onset of the light period. Plants were fumigated with 10 ppm NO_2_ for 1 h and 2 days later with 30 ppm NO_2_. 10 ppm NO_2_ had no visible effect on Col-0 (mutant background) and most mutants. However, exposure to 30 ppm NO_2_ caused dead leaf areas or complete leaf collapse in most of the tested plants. Sensitive plants displayed symptoms already after 10 ppm NO_2_ whereas tolerant plants were hardly affected even by 30 ppm NO_2_.

### Imaging

UV-induced fluorescence was detected using a hand-held UV lamp (Blak-Ray B-100AP; UVP). Plants were photographed with a Nikon DC300 digital camera.

### Sampling of candidate mutants, and DNA extraction

Mutants showing NO_2_ phenotypes were sampled at 48 h after fumigation when symptoms became clearly visible due to bleaching of the dead leaf areas. Mutants that showed obvious phenotypes compared to the overall appearance of the fumigated seedlings were selected based on the assumption that the vast majority of mutants does not have an NO_2_ phenotype different from the Col-0 background line. NO_2_ sensitive and tolerant mutant seedlings without roots were sampled into polypropylene tubes containing 10 to 12 glass beads (1.7–2.0 mm, Roth) and were immediately frozen in liquid N_2_. The sampled seedlings were homogenized twice for 10 s in a Silamat S6 bead mill (Ivoclar Vivadent). DNA was extracted using the DNeasy Plant Mini kit (Qiagen) and quantified by Quant-iT Picogreen dsDNA (Life Technologies) according to the manufacturer’s instructions.

### Adapter ligation-mediated PCR

Genes containing T-DNA insertions were identified by adapter ligation-mediated PCR following a published protocol [6]. In brief, genomic DNA from mutant plants was digested with the *Ase*1 restriction enzyme, and Ase adapters (long strand adapter 2 plus short strand of adapter Ase) were ligated to the cut sites. Fragments containing T-DNA were selectively amplified by LBa1/AP1 primer pairs binding T-DNA as well as adapter sequences. Subsequently, a nested PCR with LBb1/AP2 primers was run to selectively amplify T-DNA/gDNA junctions before sequencing. According to O’Malley et al. (2007) this step can be omitted when using Ase adapters but we found that the additional PCR enhances sequencing quality and success rate.

### T-DNA-specific target enrichment and next generation sequencing

Genomic DNA from 59 (Pool I) or 65 (Pool II) plants was pooled to give two samples each containing 1 μg DNA. The DNA was precipitated by mixing with 1/10 sample volume of 3 M sodium acetate, pH 5.2, and 2 volumes of 100% cold ethanol. Samples were then incubated at − 20 °C for at least 20 min. After centrifugation at maximum speed the resulting pellet was washed once with 1 mL 70% ethanol, briefly air-dried, and finally resuspended in 100 μl of Tris-EDTA (TE) buffer. Two libraries were prepared from the DNA pools I and II using the GS-FLX+ Rapid Library (Roche) according to the manufacturer’s instructions, which involved shearing of the DNA, fragment end repair, and adapter ligation. Small fragments were removed by AMPure XP beads (Beckman), and the appropriate length distribution of DNA fragments between 500 and 1500 bp was confirmed by measurements with the Bioanalyzer 2100 (Agilent). Libraries I and II were amplified by ligation mediated PCR using adapter-specific Rapid-A and -B primers as described in the NimbleGen SeqCap EZ Library LR User’s Guide v2.0 [25], and DNA was quantified with the Quant-iT Picogreen dsDNA assay (Life Technologies).

DNA fragments containing T-DNA were isolated from the amplified libraries using T-DNA border-specific 70mer probes, the SeqCap EZ Hybridization and Wash Kit (Roche Nimblegen), and SeqCap EZ Developer Reagent (Roche Nimblegen). The following biotinylated 70mer probes designed to bind T-DNA left- and right border (LB/RB) sequences of the Agrobacterium tumefaciens vector pROK2 were employed for target enrichment: LB1: GAG CTG TTG GCT GGC TGG TGG CAG GAT ATA TTG TGG TGT AAA CAA ATT GAC GCT TAG ACA ACT TAA TAA C; LB2: TAT ATT GTG GTG TAA ACA AAT TGA CGC TTA GAC AAC TTA ATA ACA CAT TGC GGA CGT TTT TAA TGT ACT G; LB3: CAA CAG CTG ATT GCC CTT CAC CGC CTG GCC CTG AGA GAG TTG CAG CAA GCG GTC CAC GCT GGT TTG CCC C; RB1: CTC CGC TCA TGA TCA GAT TGT CGT TTC CCG CCT TCA GTT TAA ACT ATC AGT GTT TGA CAG GAT ATA TTG G; RB2: CAG TGT TTG ACA GGA TAT ATT GGC GGG TAA ACC TAA GAG AAA AGA GCG TTT ATT AGA ATA ATC GGA TAT T; RB3: GGG TAA ACC TAA GAG AAA AGA GCG TTT ATT AGA ATA ATC GGA TAT TTA AAA GGG CGT GAA AAG GTT TAT C.

Following the NimbleGen SeqCap EZ Library LR User’s Guide v2.0 a hybridization mixture was prepared including among others 1 μg amplified library DNA and the six different 70mer probes. The latter were adjusted to 3.75 × 10^6^ molecules of each oligonucleotide in a total volume of 4.5 μl water, replacing the SeqCap EZ Library in the User’s Guide [7]. The hybridization mixture was incubated at 95 °C for 10 min and then at 47.5 °C for 40 h [7]. DNA fragments bound to the biotinylated probes were captured with Dynabeads M-270 streptavidin (Invitrogen) and amplified using 454 Rapid-A and -B primers. Target enrichment was verified by qPCR with the T-DNA right border-specific primers T-DNA_R_Rev CTG TGG TTG GCA TGC ACA TAC and T-DNA_R_For AGA TTG TCG TTT CCC GCC TT. Small fragments, primers, and primer dimers were removed with AMPure XP beads, the fragment length distribution was checked by Bioanalyzer 2100 (Agilent), and DNA concentration was determined with the Quant-iT Picogreen dsDNA assay (Life Technologies). Emulsion PCR, emulsion breaking, and sequencing using a 454 Genome Sequencer FLX instrument were performed as described in the NimbleGen SeqCap EZ Library LR User’s Guide v2.0 [26].

As Roche stopped its service for GS FLX+ sequencing in 2017, future studies will be performed using the Illumina MiSeq platform (Illumina). Here, the TruSeq DNA sample prep kit (Illumina) will be applied for sample preparation as described by the manufacturer. The read length of Illumina sequencing is in the range of 250 bases per read, if the paired end modus is used. Illumina sequencing has previously been reported to be applicable for targeted genome sequencing [7].

### Bioinformatic analysis and T-DNA insertion line identification

Bioinformatic analysis was performed using the CLC Genomics Workbench 11 software (Qiagen). Two datasets of 207,785 reads (dataset 1) and 154,847 reads (dataset 2) were used for the identification of T-DNA insertion sites. To identify T-DNA-containing sequences all reads were blasted against the T-DNA insertion vector pBIN-pROK2 using the BLASTN function under default settings. By filtering the data for high-scoring segment pair (HSP) length ≥ 30 and ≤ 343 bp and an e- value ≤5,72*E^− 11^ unspecific hits and parts of “only T-DNA” hits were filtered out. Then the reads were further trimmed with the following settings in the CLC Genomic Workbench: read length > 40 bp, low quality limit of 0.05, max. 2 ambiguous bases. Finally, one nucleotide on the 5′ terminus and adapter sequences were cleaved off and vector sequences from the vector pBIN-pROK2 were labeled as trimmed and therefore ignored for the mapping. Afterwards the remaining reads were mapped against the Arabidopsis genome sequence. Sequence alignments were performed with the ApE-Plasmid Editor (http://jorgensen.biology.utah.edu/wayned/ape/).

### Re-screen using ion leakage- and stomatal conductance measurements

Selected candidate mutants and WT plants were grown in pots at 65–85 μmol m^− 2^ s^− 1^ light intensity, 14 h light–10 h dark cycle, 20 °C/18 °C (day/night), and 65–68% relative humidity. Three-week-old plants were used for the ion leakage measurements whereas basal stomatal conductance was determined in 4-week-old plants. A fumigation chamber housing individual plants up to one tray of plants was used for re-screening of selected candidate mutants as described recently [10, 27]. Plants were fumigated for 40 min with 30 ppm NO_2_, which caused visible symptoms in 40–60% of the leaf area in WT plants. Immediately after fumigation 2 seedlings were collected into 30 mL of deionized water, and the background water conductivity (μS cm^− 1^) was determined using a conductivity meter (GLM 020A, Greisinger Electronic). After 24 h the sample conductivities were measured and the background values were subtracted. The resulting corrected sample conductivities were normalized to their respective conductivities measured after freezing and reheating to RT (i.e. 100% conductivity). Results are given as relative ion leakage. Basal stomatal conductance was measured in untreated plants using the Leaf Porometer Model SC-1 (Decagon Devices). The measurements took place in the growth chamber at 2 h – 4 h after start of the light period.

## Supplementary information

**Additional file 1: Figure S1.** NO_2_-induced cell death is associated with UV-induced green-blue fluorescence. **a.** Fumigation with 30 ppm NO_2_ for 1 h caused partial leaf collapse as visualized under white light (lower panel) and UV-induced emission of green-blue fluorescence in dying leaf areas (upper panel). Pictures were taken immediately after the fumigation. **b.** Untreated plants show blue fluorescence under UV illumination.

**Additional file 2: Table S1.** Results of sequencing after adapter ligation-mediated PCR.

**Additional file 3: Figure S2.** The identified T-DNA insertion site in the gene *AT2G16630* corresponds to the mutant line SALK_042357. **a.** Sequence reads from dataset 1 (red color) map to a T-DNA insertion in the 5′ UTR region of *AT2G16630* (yellow color represents the coding sequence (CDS); blue color represents the gene sequence). One sequence read mapped to the left and 2 reads mapped to the right border of the T-DNA insertion (bold red lines). The T-DNA left border/genomic DNA junction of the mutant line SALK_042357 (see http://signal.salk.edu/cgi-bin/tdnaexpress? JOB = TEXT&TYPE = DATA&QUERY=SALKseq_042357.1) is indicated in green color. **b.** The SALK_042357 sequence and the mapped sequencing read from our study are identical, except for one base. Screenshots from CLC Genomics workbench.

**Additional file 4: Figure S3.** Visible NO_2_-induced symptoms of the re-screened mutants. **a.** Basal ion leakage is similar between WT and the re-screened candidate mutants (*n* = 3). **b.** Plants before and **c.** at 72 h after treatment with 30 ppm NO_2_ for 40 min.

## Data Availability

The datasets used and/or analysed during the current study are available from the corresponding author on reasonable request.
