# Supplementary figures and images for: A T-DNA mutant screen that combines high-throughput phenotyping with the efficient identification of mutated genes by targeted genome sequencing
Source: BMC Plant Biol. 2019 Dec 4;19:539. doi: 10.1186/s12870-019-2162-7 (PMC6894221; doi:10.1186/s12870-019-2162-7)

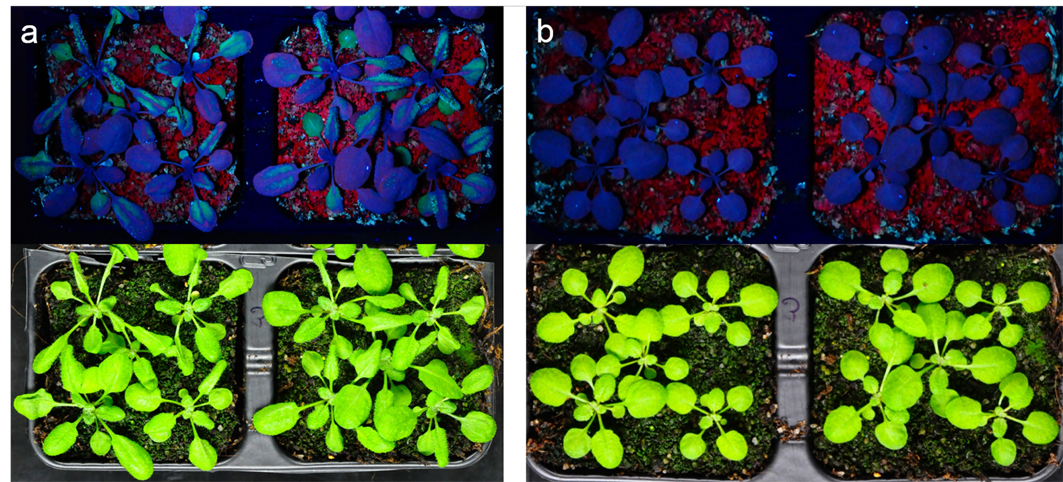

Supplement: Supplementary file 1 — Additional file 1: Figure S1. NO2-induced cell death is associated with UV-induced green-blue fluorescence. a. Fumigation with 30 ppm NO2 for 1 h caused partial leaf collapse as visualized under white light (lower panel) and UV-induced emission of green-blue fluorescence in dying leaf areas (upper panel). Pictures were taken immediately after the fumigation. b. Untreated plants show blue fluorescence under UV illumination. [file 12870_2019_2162_MOESM1_ESM.tif]

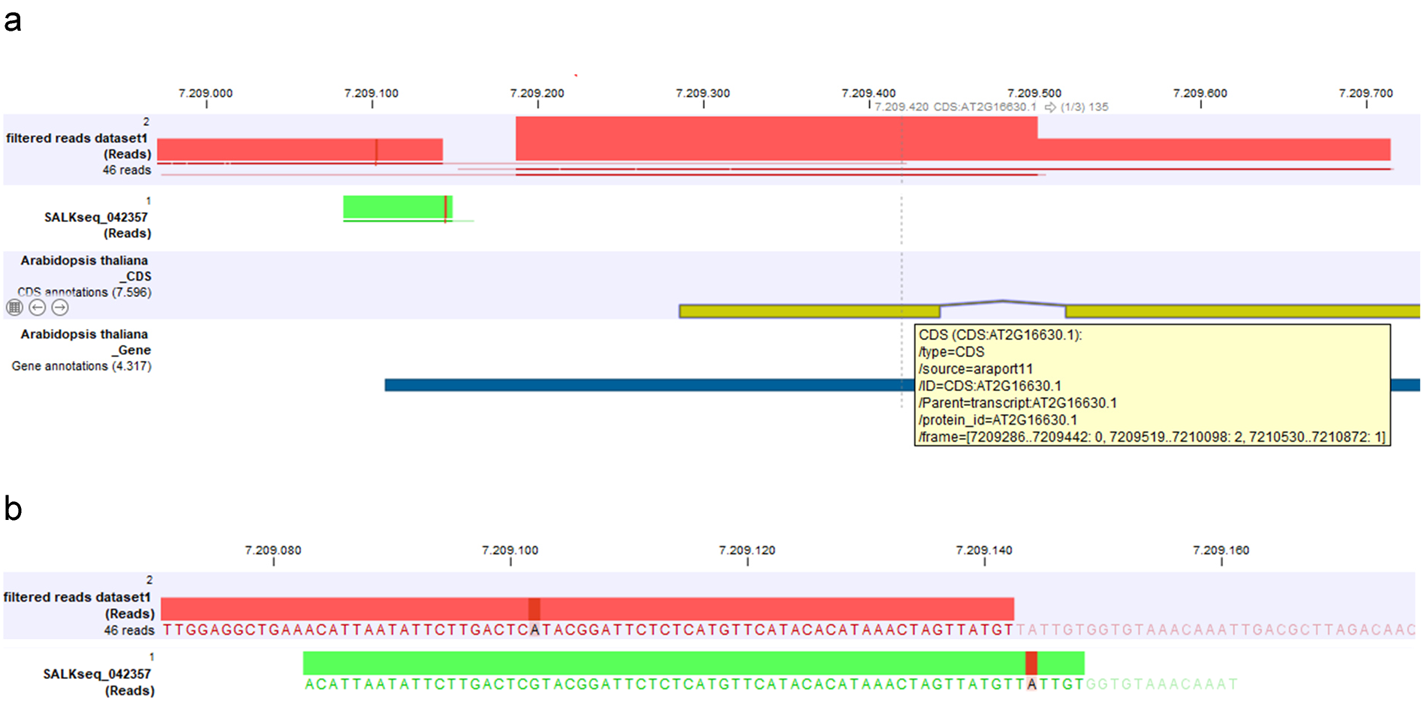

Supplement: Supplementary file 3 — Additional file 3: Figure S2. The identified T-DNA insertion site in the gene AT2G16630 corresponds to the mutant line SALK_042357. a. Sequence reads from dataset 1 (red color) map to a T-DNA insertion in the 5′ UTR region of AT2G16630 (yellow color represents the coding sequence (CDS); blue color represents the gene sequence). One sequence read mapped to the left and 2 reads mapped to the right border of the T-DNA insertion (bold red lines). The T-DNA left border/genomic DNA junction of the mutant line SALK_042357 (see http://signal.salk.edu/cgi-bin/tdnaexpress? JOB = TEXT&TYPE = DATA&QUERY=SALKseq_042357.1) is indicated in green color. b. The SALK_042357 sequence and the mapped sequencing read from our study are identical, except for one base. Screenshots from CLC Genomics workbench. [file 12870_2019_2162_MOESM3_ESM.tif]

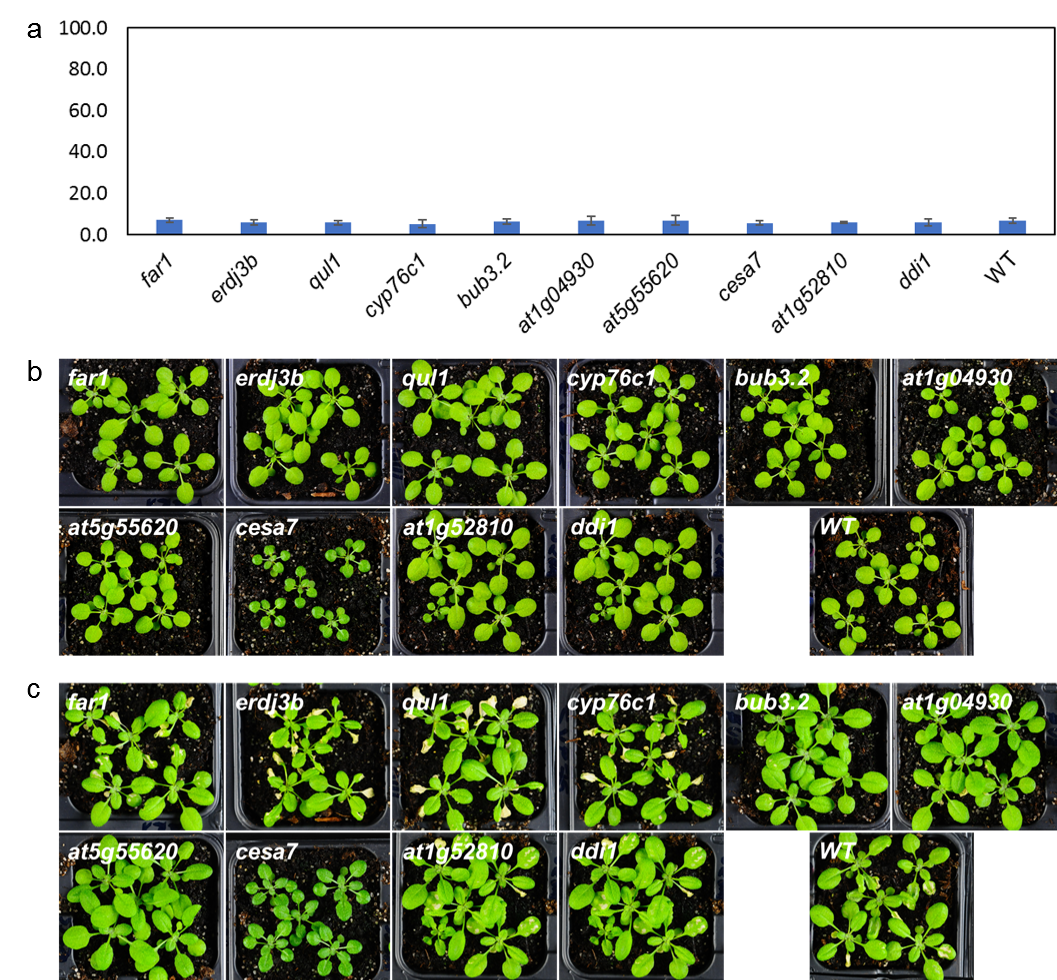

Supplement: Supplementary file 4 — Additional file 4: Figure S3. Visible NO2-induced symptoms of the re-screened mutants. a. Basal ion leakage is similar between WT and the re-screened candidate mutants (n = 3). b. Plants before and c. at 72 h after treatment with 30 ppm NO2 for 40 min. [file 12870_2019_2162_MOESM4_ESM.tif]
