# Supplementary material for: A T-DNA mutant screen that combines high-throughput phenotyping with the efficient identification of mutated genes by targeted genome sequencing
Source: BMC Plant Biol. 2019 Dec 4;19:539. doi: 10.1186/s12870-019-2162-7 (PMC6894221; doi:10.1186/s12870-019-2162-7)
Supplement: Supplementary file 2 — Additional file 2: Table S1. Results of sequencing after adapter ligation-mediated PCR. [file 12870_2019_2162_MOESM2_ESM.doc]

**Additional File 2: Table S1** Results of sequencing after adapter ligation-mediated PCR.

|  | Full-length consensus sequences; T-DNA sequences are highlighted in yellow. |
| --- | --- |
| Mutant 1 | ATCAGCTGTTGCCCGTCTCACTGGTGAAAAGAAAAACCACCCCAGTACATTAAAAACGTCCGCAATGTGTTATTAAGTTGTCTAAGCGTCAATTTGTTTACACCACAATATATCCTGACCTACCTGCCTGCCTCTATATATATACTAGTCTTTGGTCGCCTTTACTATAACCAAACATCGAGCTTGCCTTAGAGTTTGTCTCAAGCTTCTCCTTTTTTGGTATTTTGTCTTAAGCTTCCTTGCTTCTCTTTCTTCTTCTTCTCTACTCTTTCTCTCTATTTCAAACCTTCGTTTTCTTTCCTTTTTTGTATAAAATCTTAAAATCAACCAAGACAATGGAAATGAAATTGCTCTTTACATCGGCTTTCACCCAAATGTTCGGCTACTCGAACCATATGGATCAAGCCAGCAACTGCCAAAGCACCCGCAACAAAATTATAAAGATGATGAAGAAAGAAGAATTCCCAAGTGGATTCCAAGTCCCTCTTCACTACCCTAAATACTCCAAGTCTGATTACGAAGTTATGGATGATCTCCGCCTTGACTTGCTCCTCAAACAATATGGATTCTCCTTTGAAGGATCTCTTGAAGACAAGAGGGTTTTTGCAATTGAATCATTTCTCTGGCCTGATCAGCTTTAGATGGTCTATGTTGGTGTTGAATGATCTTATATCGCTCTCCACATGCATATCAATGAGCCTATACTCGAGTGGTTGAATGTTTTTAAAATTATGTCAAATTACCATGTATATCAATCACTTAGTAATGTTTTAGAACTATGTCAAATACTAGTGTGTGTTATATTTCTTTGTGTGTGGGGATGTTTTGAAGTATGCAATTGTCAAATACTAGTGTGTGTTATATTTCTTTGTGTGTGGGGATGTTTTGAAGTATGCAATTCTTCACATATATATGTATCATATGAGTGCATTAGCACAGCCCGCCGCCGTCTCGCACAAA |
| Mutant 2 | CTCAATGCGCGGTGAGGCATCAGCTGTTGCCCGTCTCACTGGTGAAAAGAAAAACCACCCCAGTACATTAAAAACGTCCGCAATGTGTTATTAAGTTGTCTAAGCGTCAATTTGTTTACACCACAATATATTGTCCGTCTAAAGACTGTGAACGGGATGCCTATATGGAAAGCAGAAAATGATACCCTTGGATTTCAAGTCCAATTAGCTCTAGAGAGAGGCCTTCCTGCAATGATTGGAAATTTCCTTTCAAAACAGCTTCCATATCCAGCTTTATCATTTGACATGGTCCACTGTGCTCAATGTGGGATTACTTGGGATATAAAAGGTATTTATATGGTATGATTCATAGTCTTTCACTTATTTTGGTTAAGATCTTTTTGAAATGATCTTCTTAAGACTGACAGTGTTATGCTCTCAATTTTCTGACGTTGAACAGATGCAATGCTACTTTTACAACTGGATCGTGTTCTCACGCCTGGGGGTTATTTTGTTTTGACCTCTCCTACAAACATTGCACTGGGAAATTCTCTTGATACTAAAAAAACAATTAACACAACACGTGTGAACGATTTATCTAATAAAATTTGCTGGAGTCTCTCAGGTCAGCCTGTGACACGTCTCTTTCGCATTAAACATGACATCCAAATTGCTACTCATCTCAATACTTAAATAAACAGAGTACGAACCTTGAATTAAACTCACATTCCCACTTAGTCTTTCTTTCTTTTTCATGGATGGTGACTCTCACACGTTCC |
| Mutant 3 | ATCAGCTGTTGCCCGTCTCACTGGTGAAAAGAAAAACCACCCCAGTACATTAAAAACGTCCGCAATGTGTTATTAAGTTGTCTAAGCGTCAATTTGTTTACACCACAATAAC  ATAACTAGTTTATGTGTATGAACATGAGAGAATCCGTACGAGTCAAGAATATTAACACAGCCCGGGCCGTCGACCAAAG |
